# Gut Microbiota and Probiotics in Influenza: A Narrative Review of Mechanisms and Emerging Evidence

**DOI:** 10.3390/v18050553

**Published:** 2026-05-12

**Authors:** Feihu Guan, Jie Zhang, Ye Tian, Bofan Fu, Ji Liu, Yafen Song, Aoyang Yan, Bing Zhang, Ling Chen, Min Zhang, Pengfei Du, Lei Wang, Xiaoyue Yang, Sifan Guo, Chenghuai Yang, Hui Zhang, Qianyi Zhang

**Affiliations:** 1 National Center for Veterinary Culture Collection, China Institute of Veterinary Drug Control, Beijing 102629, Chinaxiaoyue_yang2025@126.com (X.Y.);; 2 College of Animal Science and Technology, Shihezi University, Shihezi 832000, China; 3 College of Veterinary Medicine, Gansu Agricultural University, Lanzhou 730070, China

**Keywords:** gut microbiota, influenza virus, probiotics, gut–lung axis

## Abstract

The gut microbiota, often referred to as the “forgotten organ”, plays an indispensable role in maintaining host physiological metabolism, immune function, and nutrient absorption. Moreover, the gut microbiome serves as a critical biological barrier against viral infections and is increasingly recognized as a potential target to augment antiviral therapies. Recent studies have revealed that microbial ligands and metabolites derived from the gut microbiota are pivotal in modulating respiratory immune responses, providing compelling evidence of the complex interaction network between microorganisms and the host, particularly the signaling pathways linking the gut to distal organs such as the lungs. This review examines the communication and regulatory mechanisms between the gut microbiota and pulmonary mucosal surfaces during influenza virus infection, emphasizing how gut microbial communities and probiotics influence host immune responses, promote the production of immune-related molecules, and enhance antiviral defenses. The aim is to provide comprehensive insights into the gut–lung axis and its implications for respiratory health.

## 1. Introduction

Influenza, one of the most concerning viral pathogens within the realm of global public health, currently has no comprehensive prophylactic or therapeutic strategies that are entirely effective in mitigating influenza pandemics [1,2]. Extensive research has traditionally focused on the interactions between influenza viruses and respiratory epithelial cells, as well as the role of associated airway microbiota in disease pathogenesis, to seek new therapeutic targets [3,4,5,6]. The recent proposal of the concept of “gut–lung axis” not only facilitates the understanding of the importance of intestinal microbiota, as a key “hidden” regulatory factor in host health, for the immunoregulation of lung health, but also provides a brand-new perspective for research on respiratory immunity and pulmonary health [3,4].

The gut microbiota interacts dynamically with the host’s gut-associated lymphoid tissue (GALT)—the most abundant immune cell reservoir—beginning early in development and exerting profound influence on immune modulation [5,7,8,9]. Through the production of diverse metabolites and direct cellular interactions, the microbiota influences distal organ immunity, including the brain, lungs, and liver, thereby affecting antiviral defenses [10,11,12]. Recent shifts in research paradigms have focused on the role of the gut–lung axis in influenza pathogenesis, specifically examining how host cellular immune activation and innate defense mechanisms contribute to viral control [13]. These studies aim to clarify the association between pulmonary diseases—including pneumonia, influenza, and COVID-19—and gut microbiota composition [14,15].

Although the primary immune response to influenza virus occurs within the lungs, substantial evidence indicates that gut microbiota and their metabolic products, such as short-chain fatty acids (SCFAs), are integral to the host’s immune regulation throughout the disease course—affecting both innate and adaptive immunity [16,17,18]. Dysbiosis of the intestinal microbiota correlates with decreased protective metabolites and immune imbalance, such as shifts in regulatory T cell and Th17 cell populations, which may be associated with elevated risk or worsened severity of influenza infection, yet causal evidence remains to be fully established. It is thus established that mucosal immunity in the respiratory tract is not solely governed by local microbiota but is also significantly modulated by distal microbial communities within the gut [19]. This cross-organ immune regulation positions gut microbiota as promising targets for therapeutic strategies against respiratory and pulmonary infections [7].

Interventions employing probiotics and other microbiota-modulating agents have demonstrated promising outcomes in preclinical models and some human immunogenicity studies, highlighting the potential of microbiota-based therapies [20,21,22]. It should be noted that although the current relevant evidence is based on a variety of influenza strains, host species and challenge models, and the virus pathogenesis, tissue tropism, virulence and host immune phenotypes are different, these beneficial microorganisms support host health by maintaining gut microbial homeostasis and enhancing innate immune responses, ultimately providing a stable internal environment conducive to viral resistance [23]. Furthermore, such interventions can modulate cytokine secretion and, when combined with vaccination, boost protective antibody responses, thereby facilitating viral clearance during infection. These approaches underscore the vital role of gut microbiota in adaptive immunity regulation and emphasize their potential as adjunct therapies to improve influenza vaccine efficacy and overall disease outcomes [24].

This review article delineates the current evidence linking influenza virus infection to alterations in the gut microbiota within human and animal models. It provides a comprehensive overview of recent advances in understanding the role of intestinal microbial communities in mediating antiviral immune responses to influenza, with particular emphasis on the underlying mechanisms of innate and adaptive immunity. Furthermore, it discusses the impact of probiotic interventions on gut microbiota composition during influenza infection and their modulatory effects on antiviral immunity. Overall, this review offers a mechanistic overview and highlights emerging but still preliminary evidence for microbiota-targeted approaches, with a view to providing a theoretical framework and research perspectives for future development of innovative interventions and prophylactic preventive strategies targeting the influenza virus through microbiota modulation.

## 2. Literature Selection Approach

This manuscript was prepared as a narrative review intended to provide a focused and up-to-date overview of current evidence regarding the interactions between influenza virus infection, gut microbiota alterations, microbiota-mediated antiviral immunity, and probiotic-based interventions. A comprehensive literature search was conducted using major electronic databases, including PubMed, Web of Science, and Scopus. The search covered publications from database inception to March 2026 (last search date: 15 March 2026). The search strategy focused on publications related to influenza virus infection, gut microbiota, the gut–lung axis, antiviral immunity, and probiotic interventions, using combinations of keywords such as “influenza,” “gut microbiota,” “intestinal microbiota,” “gut–lung axis,” “microbiome,” “antiviral immunity,” “influenza vaccine,” “clinical trial,” “randomized controlled trial,” and “probiotics.” In addition to mechanistic and preclinical studies, particular attention was paid to the identification of human clinical studies, including randomized controlled trials and other clinical studies, as well as previously published review articles, systematic reviews, and meta-analyses relevant to this topic.

Eligible studies included peer-reviewed original research articles and review articles published in English that were directly relevant to influenza-associated gut microbiota alterations, microbiota-mediated antiviral immune responses, or microbiota-targeted interventions for influenza prevention and control. Seminal earlier studies were also included where necessary to provide essential background and mechanistic context. Additional relevant publications were identified through manual screening of the reference lists of key articles. Studies not directly related to the topic, conference abstracts, editorials, and articles lacking sufficient relevance or methodological clarity were excluded. Because the aim of this manuscript was to provide a focused narrative overview of current evidence, a formal systematic review protocol and PRISMA-based study selection process were not applied.

## 3. Bidirectional Regulation and Immune Modulation of Gut Microbiota in Influenza Virus Infection

The gut microbiota and the host constitute a complex micro-ecological system, comprising phyla such as *Firmicutes*, *Bacteroidetes*, *Proteobacteria*, *Verrucomicrobia*, *Actinobacteria*, and *Cyanobacteria*. The human gut microbiota comprises approximately 10^14^ microbial cells and encompasses a highly diverse community of bacteria. As a core component of the host’s microbial ecology, it is closely associated with host health, playing a critical role in maintaining intestinal mucosal integrity, modulating immune responses, synthesizing nutrients and metabolic products, and defending against pathogenic microorganisms [25].

**Figure 1:**
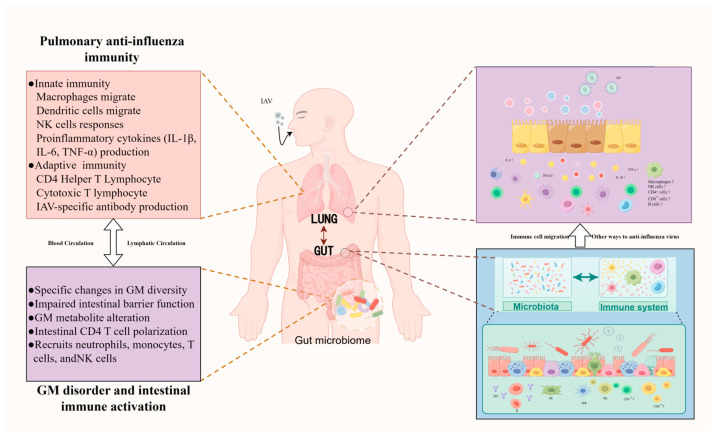
Dynamic interactions between the lung and intestinal microbiota during IAV infection (created with FigDraw 2.0 (by figdraw.com)). Following IAV infection, the activation of the host’s innate immune response in the lungs and the excessive secretion of inflammatory cytokines in the body disrupt intestinal homeostasis, leading to intestinal microbiota dysbiosis and intestinal barrier impairment. Consequently, the production of signaling molecules derived from the intestinal microbiota is reduced. These molecules enter the circulation to activate intestine-associated immune pathways, which can stimulate and recruit immune cells such as CD4^+^ T cells and CD8^+^ T cells. A gut–lung crosstalk is established through the blood and lymphatic circulation, facilitating the migration of immune cells to the lungs and enhancing the lung’s defense capacity against IAV infection.

Recent clinical and animal experimental studies have demonstrated that respiratory infections caused by influenza viruses are frequently accompanied by gastrointestinal manifestations, particularly via the perturbation of gut microbiota structure and compromise of intestinal barrier integrity, indicating a complex and close association between gut microbiota and host antiviral immune responses (Figure 1) [26,27,28,29,30]. Healthy intestinal commensal bacteria interact with intestinal epithelial cells and antigen-presenting cells (e.g., dendritic cells), activating pattern recognition receptors (PRRs), including Toll-like receptors (TLRs) and NOD-like receptors (NLRs), thereby initiating signaling pathways such as NF-κB and mitogen-activated protein kinases (MAPKs) and inducing the expression of multiple immune effector molecules. This process not only promotes the differentiation of effector T cells (e.g., Th1 and Th17) but also maintains immune tolerance mediated by regulatory T cells (Tregs), thus achieving a balance between immune defense and immune homeostasis. Furthermore, metabolites produced by gut microbiota, particularly SCFAs, can regulate the expression of immune-related genes at the epigenetic level by binding to G protein-coupled receptors (GPR41 and GPR43) or inhibiting histone deacetylases (HDACs), thereby enhancing the host’s antiviral capacity.

Conversely, influenza virus infection can feed back on the gut microbiota through this axis, leading to dysbiosis characterized by a reduction in obligate anaerobes and an increase in pathogenic bacteria such as Enterobacteriaceae. Such an imbalance may compromise the intestinal mechanical and microbial barriers, resulting in a decreased expression of tight junction and adhesion proteins and facilitating translocation of bacteria and toxins into the circulation, which can trigger secondary infections and impair antiviral immunity [31,32]. Post-infection, pulmonary CCR9^+^CD4^+^ T cells secrete interferon-γ (IFN-γ), recruiting to the gut via circulation, stimulating intestinal epithelial cells to produce interleukin-15 (IL-15), and promoting Th17 cell polarization as well as heightened intestinal pro-inflammatory cytokine expression. This process not only damages the gut mucosa but also elevates the risk of systemic dissemination of other pathogens [28,33]. Additionally, influenza infection alters gut microbial metabolism, reducing SCFAs, thereby weakening microbiota-mediated immunomodulation and lowering pulmonary resistance to pathogens [34,35]. Beyond SCFAs, gut microbiota produce various metabolites such as tryptophan derivatives and bile acids, which can reach the respiratory tract via circulation and modulate immune cell activity and cytokine production with precision [36]. For instance, during influenza A virus (IAV) infection, tryptophan metabolites activate the aryl hydrocarbon receptor, suppressing excessive inflammatory responses to protect respiratory tissues. Similarly, acetate exerts antiviral effects in both human ex vivo lung models and lung epithelial cells by blocking viral entry and modulating cellular metabolic status and antiviral responses [37]. While bile acids can regulate macrophage phagocytic activity, enhancing viral clearance [38,39]. Studies have demonstrated that broad-spectrum antibiotic exposure triggers profound microbial dysbiosis, resulting in a nearly 1000-fold decrease in host serum secondary bile acids. Such metabolic perturbations are tightly linked to the activation of AP-1/NR4A-driven proinflammatory signaling cascades and inflammasome assembly [40]. Additionally, the disruption of lung–intestinal immune homeostasis caused by antibiotic overuse not only significantly increases the mortality rate of IAV infection but also markedly impairs the therapeutic efficacy of antiviral drugs [41]. These mechanisms suggest that, although IAV does not directly invade the gut, it does so via cross-mucosal immune interactions mediated by the mucosal axis, providing a pathway through which influenza influences gastrointestinal physiology, ultimately increasing susceptibility to disease progression and other severe complications.

Furthermore, gut microbiota can activate the intestinal mucosal immune system, promoting the maturation and activation of immune cells such as macrophages and dendritic cells. These cells rapidly migrate to the respiratory mucosa during infection, participating in viral recognition and clearance [35]. For example, *segmented filamentous bacteria* (SFB) induce the formation of resident alveolar macrophages (AMs) in the mouse lungs, enhancing their phagocytic capacity and stimulating the secretion of complement component C1qa, thereby effectively neutralizing influenza viruses and other respiratory pathogens, which reduces infection risk [18,42]. Intestinal colonization by SFB also programs alveolar macrophages to preferentially utilize oxidative phosphorylation and complement-dependent phagocytosis, enabling efficient defense against and killing of secondary bacterial infections caused by *Streptococcus pneumoniae*, Haemophilus influenzae, or Staphylococcus aureus [43]. Additionally, through immune cell trafficking and cytokine signaling, gut microbiota may exert feedback regulatory effects on pulmonary antiviral responses, underscoring their pivotal role in host defense against influenza virus infection.

Studies in germ-free and antibiotic-treated mice conducted by Ngo et al. demonstrate that the composition of gut microbiota significantly influences host resistance to influenza virus infection [35]. Antibiotic-mediated depletion of the gut microbiome in germ-free mice (EF mice) compared to specific pathogen-free (SPF) mice results in heightened hypothermia and weight loss upon intranasal inoculation with H1N1 influenza A virus. The underlying mechanism involves the impaired activation of innate and adaptive immune responses in EF mice, characterized by insufficient pulmonary CD4^+^ and CD8^+^ T cell responses and reduced antiviral gene expression in alveolar macrophages. Furthermore, germ-free mice exhibit exacerbated pulmonary inflammation, higher viral titers, and increased mortality following H1N1 infection [44].

These effects are attributed to dysbiosis-induced disruption of intestinal barrier integrity, permitting translocation of endotoxins and other harmful substances into systemic circulation, thereby triggering systemic inflammatory responses that disturb respiratory immune homeostasis and ultimately weaken antiviral defenses. The findings highlight that the stability of the gut microbiota directly governs immune cell development and activation; microbiota depletion and imbalance markedly elevate host vulnerability to influenza infection by impairing the generation of pulmonary T lymphocytes, compromising adaptive immunity, escalating viral load, and exacerbating lung tissue damage [18,42]. Similar detrimental effects have been observed in humans infected with H7N9 influenza; gut microbiota dysbiosis is associated with more severe disease and longer recovery, indicating a consistent role of gut microbiota in modulating host responses [18,42].

Moreover, microbiota-derived metabolites such as SCFAs enhance antigen presentation by dendritic cells, thereby promoting adaptive immune responses [45]. The microbiome also facilitates T and B cell differentiation and proliferation, augmenting the production of virus-specific antibodies and effectively suppressing viral replication and dissemination [46,47]. Variations in microbiota composition are associated with differences in host susceptibility and disease progression post-infection. Notably, influenza virus infection not only promotes the overgrowth of pathogenic gut bacteria but also creates a favorable environment for opportunistic respiratory microbiota, which can further compromise host defenses. Multi-omics analyses reveal that reductions in SCFAs during influenza infection are linked to decreased abundance of *Helicobacteraceae* and *Clostridiales*, weakening immunomodulation and elevating the risk of secondary pulmonary infections. Valentin Sencio et al. further confirmed that influenza-induced intestinal dysbiosis impairs resistance to *Streptococcus pneumoniae*, increasing susceptibility to secondary pneumonia. Additional studies indicate strain-specific effects of influenza viruses on gut microbiota, with H1N1 strains such as A/Fort Monmouth/1/1947 (FM1) and A/Puerto Rico/1934 (PR8) eliciting distinct alterations in microbial community composition, likely due to differences in viral pathogenicity and host environment, thereby affecting immune response intensity and disease outcomes.

In summary, the gut microbiota plays a multifaceted role in modulating host immune responses during influenza virus infection through various mechanisms, including immune regulation, barrier integrity maintenance, metabolic influence, and mucosal immune axis modulation. These interactions facilitate the activation, proliferation, and targeted migration of immune cells to the site of infection, establishing a bidirectional gut–lung regulatory axis (Figure 1). Although it is difficult to directly extrapolate the mechanisms elucidated based on a single strain or model to other systems due to differences in virus strains, host species, and experimental conditions, an in-depth analysis of the above-mentioned mechanism correlations can not only provides theoretical support for regulating the intestinal microbiota and developing influenza intervention strategies based on the intestinal microbiome to enhance the body’s antiviral defense, but also provides scientific rationale for the advancement of novel influenza vaccines.

## 4. Probiotic Interventions Against Influenza Infection: Evidence and Potential

Probiotics are a class of microorganisms that, when administered in sufficient quantities, confer beneficial effects on host health. As preventive potent agents, they effectively modulate intestinal microbiota diversity, community structure, and metabolic activity, and exhibit immunomodulatory and antiviral potential in preclinical models [21,48]. Upon entering the gut, probiotics participate in immune regulation through mechanisms such as microbiota equilibrium, reinforcement of intestinal epithelial barrier function, and inhibition of pathogenic microbial growth and adhesion [49,50]. A recent clinical meta-analysis indicated that oral probiotics could modestly reduce the incidence, duration, and severity of respiratory tract infections among non-elderly individuals, supporting the notion that probiotics exert immunomodulatory benefits in the context of respiratory infections [51,52,53].

A seminal study in 1999 confirmed that feeding mice with *Bifidobacterium breve* YIT4064 significantly elevated serum influenza-specific IgG levels [54]. This was the first clear evidence of probiotic benefits in influenza virus infection, pioneering new strategies for antiviral development. Subsequent research demonstrated that various probiotic strains—including Bifidobacteria, yeast, spore-forming bacteria, and Lactobacilli—exhibit positive effects in both animal models and clinical studies for host resistance against influenza [55]. These strains, despite differing mechanisms, often involve Th1 cell-mediated immune responses as a core pathway [56]. For instance, *Lactobacillus plantarum* SBT2055 (LG2055), known for its bile tolerance and gut environment modulation, upregulates macrophage Mx1 expression, induces antiviral genes such as Mx1 and Oas1a in lung tissues, and thereby inhibits viral replication and reduces pulmonary inflammation to exert anti-influenza effects [57]. The pronounced improvement in lung pathological injury achieved by combined administration of *Lactobacillus plantarum* GUANKE and tryptophan (Trp)—via modulation of cytokine profiles and restoration of epithelial barrier integrity—is mechanistically driven by microbial metabolism of Trp into the beneficial metabolite indole-3-lactic acid (ILA). ILA subsequently activates and facilitates crosstalk between the AHR/STAT3/IL-10 signaling axis, ultimately mitigating inflammatory responses [58].

In mice models, *Lactobacillus* strains can bind to CD11c^+^ immune cells in Peyer’s patches, elevating interferon (IFN) expression. This interaction increases influenza-specific IgA levels in bronchoalveolar lavage fluid (BALF), serum, small intestine, and lungs, alleviating pulmonary pathology and improving survival post-influenza infection. Additionally, *Lactobacillus reuteri* 1025 significantly mitigates weight loss, alleviates symptoms, reduces viral titers in infected mice, and restores the relative abundance of *Firmicutes* and *Bacteroidetes* in the gut [59]. *Short bifidobacteria strain* CCFM1026 modulates immune responses by decreasing neutrophil ratios, increasing lymphocyte counts, regulating TLR7, MyD88, TRAF6, and TNF-α expression, and adjusting the *Bacteroidetes* to *Firmicutes* ratio within the gut microbiota, thus correcting immune dysregulation. Furthermore, probiotic treatment has been shown to suppress Th2-mediated immune responses in mice, downregulating cytokines such as IL-6, IL-4, IL-5, and IL-10, which may directly attenuate influenza-related pulmonary inflammation [60]. Of note, *Lactobacillus* murinus colonization mitigates intestinal microbial dysbiosis following IAV infection and lowers the risk of secondary MRSA infection by enhancing T cell-independent IgA production [61].

Recent studies reveal that probiotic flagellin components promote retinoic acid synthesis, facilitating IgA^+^ B cell differentiation and strengthening mucosal responses against influenza viruses [62]. Additionally, factors involved in IgA production—such as TNF, B cell-activating factor (BAFF), and inducible nitric oxide synthase (iNOS)—may synergistically contribute to intestinal microbiota-mediated resistance to influenza infection [63]. Animal experiments demonstrate that probiotic intervention in mice results in significant increases in total and influenza-specific IgA levels in BALF and serum, correlating with improved survival rates following influenza virus challenge [64].

Regarding specific probiotic strains, *Lactobacillus plantarum* B240 enhances immune function of mice by increasing the secretion of IgA and IgG, thereby exerting anti-influenza effects [65]; *Lactobacillus plantarum* 0111 interventions can restore *Lactobacillus* and *Bacteroides* populations diminished by H9N2 influenza infection in mice, maintain gut microbiota homeostasis, and stimulate upregulation of IFN-β, interferon-stimulated genes (ISGs), and IgA. This modulation of intestinal microbiota-mediated innate and adaptive immune responses enhances protection against the influenza virus and highlights its potential as a probiotic for influenza prevention and therapy [66]. Emerging evidence indicates that oral administration of bifidobacteria and lactobacilli strains in a mouse influenza model confers protective effects via augmentation of humoral immunity, characterized by decreased pro-inflammatory cytokines or increased antiviral cytokines in lung tissue, Peyer’s patches, bronchoalveolar lavage fluid (BALF), blood, mediastinal lymph nodes, and spleen cells [67]. For example, oral *Bifidobacterium breve* confers protection by inducing serum anti-influenza IgG antibody production, while oral *Enterococcus faecium* reduces disease severity and pulmonary inflammation via modulation of monocyte chemokine CCL2/C-C motif chemokine receptor 2 (CCR2) signaling pathway [68,69]. Notably, probiotics have been confirmed to enhance natural killer (NK) cell activity, reduce pulmonary granulocyte counts, and increase Th1-associated cytokines during influenza virus infection, thus offering novel strategies for viral control [64,70,71]. Although variations in probiotic strains, influenza viral subtypes, and dosing regimens may lead to differing outcomes, these do not undermine the pivotal role of microbiota modulation in anti-influenza immunity. Both viable and non-viable probiotics can interact with host pattern recognition receptors (PRRs) via microbial-associated molecular patterns (MAMPs) on cell walls, transmitting signals through Toll-like receptors (TLRs) and C-type lectin receptors to regulate cytokine expression, enabling the immune system to accurately detect pathogen invasion and initiate appropriate immune responses to maintain immune homeostasis [72]. In summary, these studies suggest that combined use of probiotics (Table 1) during influenza infection and vaccination may yield synergistic or enhanced effects, with limited support from human immunogenicity studies (Table 2) and few strains reaching clinical endpoint for influenza treatment. And there is currently no probiotic strain that has not shown stable and consistent efficacy in different influenza subtypes (H1N1, H9N2, etc.) and animal models. Future research should optimize probiotic strain selection, dosing strategies, timing, and treatment duration to resolve existing discrepancies and further substantiate the clinical utility of probiotics in influenza management.

## 5. Probiotics and Mucosal Immunity in Influenza: Mechanistic Insights

Mucosal immunity constitutes the primary interface between the host and the external environment and includes both gastrointestinal and respiratory compartments. The interaction between immune cells in the mucosal tissues of the respiratory tract and gastrointestinal tract constitutes the “mucosal immune axis”, in which immune cells activated in a certain mucosal tissue can migrate to distant mucosal sites and exert their effects [118,119]. Within this framework, gut-associated lymphoid tissue (GALT) and bronchus-associated lymphoid tissue (BALT) coordinate immune responses, regulate innate and adaptive immune responses through neural, endocrine, and immune pathways, as well as mucosal immune responses that play an important role in host defense against influenza viruses [120]. Secretory immunoglobulin A (sIgA), as a key effector molecule of mucosal immunity, plays a key role in neutralizing pathogens, including the influenza virus, by preventing virus attachment and invasion of epithelial cells. Therefore, the regulation of mucosal immunity is a key mechanism by which intestinal flora and probiotics affect respiratory viral infections [121].

Probiotics are live microorganisms that confer health benefits when administered in adequate doses and are capable of modulating host immunity in a manner that mimics or enhances the functions of the endogenous microbiota. Numerous studies have demonstrated that specific probiotic strains can potentiate mucosal immune responses, including the promotion of secretory immunoglobulin A (sIgA) production and the improvement of intestinal barrier function [122]. Evidence from human immunogenicity and vaccine-response studies (Table 2) indicates that probiotic interventions may enhance immune parameters, including influenza vaccine-specific antibody titers, T cell responses, and mucosal IgA production. Furthermore, probiotics can regulate innate immune signaling pathways by activating pattern recognition receptors, thereby inducing the secretion of antiviral cytokines (e.g., type I interferons). Emerging evidence also suggests that probiotics may induce trained immunity, which in turn enhances the host’s basal immune response to viral infections [123]. Notably, these immunomodulatory effects are highly strain-specific and may vary depending on host-related factors and experimental conditions.

On this basis, in addition to the classic innate immunity and adaptive immune responses, the “trained immunity” proposed in recent years provides a new theoretical framework for understanding the systemic immune regulation of intestinal microbiota [124]. Gut microbiota-derived signals, including SCFAs and microbe-associated molecular patterns, have been shown to influence the training of innate immune cells, thereby enhancing antiviral responses. Intestinal microbiota and probiotics can continuously stimulate bone marrow progenitor cells and peripheral innate immune cells through their structural components (lipopolysaccharide, peptidoglycan, etc.) and metabolites (SCFAs, secondary bile acids, etc.), induce histone modifications (such as H3K4me3 and H3K27ac.) and changes in chromatin open state, thereby enhancing the rapid expression of inflammatory factors (IL-6, TNF-α, etc.) and antiviral molecules (type I interferon, etc.) [125]. In addition, specific probiotics (*Lactobacillus* and *Bifidobacterium*, etc.) have been proven to promote the enhancement of glycolysis by regulating metabolic pathways such as mTOR and HIF-1α, further supporting the maintenance of trained immune phenotypes [126,127,128]. This “preactivation” state enables the host to more quickly and effectively initiate an early immune response when encountering respiratory pathogens such as influenza viruses.

On the one hand, activated or “trained” immune cells (including T cells, B cells and functionally remodeled innate immune cells) in the intestine can migrate to the lung tissue through lymph and blood circulation to enhance local immune defense [129,130]; on the other hand, microbial metabolites such as SCFAs can enter the systemic circulation and act on lung immune cells, regulating the polarization state of macrophages and the expression of antiviral genes in epithelial cells [131]. For example, butyrate can enhance the activity of the interferon signaling pathway while inhibiting excessive inflammatory responses, thereby playing a dual regulatory role between antiviral defense and inflammatory control [132]. Supplementation of specific probiotics (such as *Lactobacillus* and *Bifidobacterium*) can not only enhance mucosal sIgA secretion and increase natural killer cell (NK cell) activity, but can also enhance early antiviral responses by inducing trained immunity, thereby reducing the severity of influenza infection to a certain extent [130]. In addition, healthy intestinal flora keeps the body in a functional “early warning” state by maintaining basal levels of interferon signaling and trained immune status, helping to quickly initiate immune responses in the early stages of viral infection [133]. Probiotic intervention may also be involved in training immune processes by modulating host–microbe interactions and promoting a state of immune readiness [134]. Although most of the current evidence comes from experimental models, these findings suggest that microbiota-induced trained immunity may be an important mechanism for host resistance to influenza virus infection. However, further clinical studies are still needed to verify its effects in humans.

Overall, the gut microbiota and probiotics influence influenza virus infection through multiple interrelated mechanisms. These mechanisms include enhancing mucosal barrier function, regulating innate and adaptive immune responses, and modulating inflammatory processes. Through the gut–lung axis, the above effects can extend to the respiratory tract, thereby changing the process of viral replication, immune activation and tissue damage in the respiratory tract. Although existing preclinical evidence fully clarifies the potential mechanism by which probiotics mediate immune training and strengthen the body’s influenza defense capabilities, there is still a significant gap between basic research results and clinical practical applications, and the translation value is limited. Heterogeneity in probiotic strains, host characteristics, and study designs results in inconsistent results from human studies. Therefore, further research is still needed to clarify the clinical value of microbiota-targeted interventions in the prevention and treatment of influenza.

## 6. Probiotic Vector-Based Vaccines for Influenza: Current Evidence and Translational Limitations

The respiratory mucosal epithelium is regarded as the primary line of defense against influenza virus infection, occupying a central role within the host’s immune defense system [135]. Empirical evidence indicates that effective mucosal vaccination can elicit cross-protective immune responses against multiple influenza virus subtypes [136]. Consequently, enhancing mucosal immune function to combat influenza has emerged as a critical strategic focus and a pivotal scientific challenge in influenza prevention and control. Recent advances in vaccine development emphasize novel formulations capable of inducing antigen-specific immune responses at mucosal surfaces; such vaccines not only activate localized mucosal immunity but also stimulate systemic immune responses, thereby playing a significant role in reducing viral transmission and disease incidence [137]. Given the biological properties of probiotics—such as specific mucosal adhesion, superior antigen targeting delivery, and precise immune modulation—they demonstrate unique advantages in preventing mucosal infections. Developing ‘universal’ vaccines using probiotics as vectors that encompass multiple influenza virus subtypes is a promising intervention, increasingly recognized as a key direction in vaccinology research [22].

The strategic development of universal influenza vaccines primarily involves targeting conserved antigenic epitopes, notably the extracellular domain of matrix protein 2 (M2e) and the stalk region of hemagglutinin (HA) [138]. M2e-specific antibodies can mediate robust immune protection through Fc receptor (FcR)-dependent antibody-dependent cellular phagocytosis (ADCP) and antibody-dependent cellular cytotoxicity (ADCC) [139]. Meanwhile, multi-epitope fusion design of the conserved HA stalk region enables the activation of both humoral and cellular immune responses—‘antibody-mediated’ and ‘T cell-mediated’ immunity—substantially extending the duration and breadth of protection [140]. Incorporating multiple subtypes of conserved HA stalk epitopes could induce dual ‘antibody plus T cell’ immune mechanisms, thereby broadening the protective scope and duration [141]. The integration of this antigenic design with the immunomodulatory capacity of probiotics offers a promising avenue to enhance vaccine efficacy. Recent studies indicate that oral administration of *Escherichia coli* Nissle 1917 (EcN), a probiotic capable of eliciting mucosal immune responses, alleviates respiratory symptoms, attenuates body weight loss, and mitigates pathological lesions in mice infected with H9N2 or H1N1 influenza A virus. Moreover, this strain can serve as a vector for recombinant vaccines expressing porcine influenza virus hemagglutinin, leading to increased expression of CD40 and CD86 in Peyer’s patches, activation of dendritic cells, and consequently the induction of potent mucosal immunity in mice—offering effective protection against heterologous influenza virus infections [142,143]. Additionally, another approach utilizing EcN to express a multivalent M2e concatemer as a secreted protein has been employed to develop intranasal vaccines; experimental results show rapid activation of innate respiratory immune defenses within 24 h post-administration and the sustained induction of high-titer respiratory tract mucosal and systemic immune responses in female BALB/c mice, providing broad-spectrum immunoprotection against diverse influenza strains [22].

In the field of vaccine development utilizing *Lactobacillus* as a delivery vector, significant advancements have been achieved through relevant studies. Findings demonstrate that genetically engineered plant-derived *Lactobacillus* expressing recombinant influenza virus M2e and hemagglutinin (HA) antigens markedly enhance intestinal mucosal IgA and serum IgG antibody responses in vaccinated animals. In murine models post-immunization, there is a notable increase in B220^+^IgA^+^ cells within Peyer’s patches (PPs), alongside heightened lymphocyte proliferation and elevated CD8^+^ T cell frequencies capable of secreting IFN-γ, conferring effective protection against H9N2 and H1N1 viral challenges [144]. Furthermore, oral administration of recombinant *Lactobacillus* expressing H5N1 HA induces HA-specific serum IgG and IgA antibodies, providing similar protective efficacy against fatal H5N1 influenza infection [145]. Parallel research utilizing other probiotic carriers corroborates these outcomes: for instance, Enterococcus L3 expressing HA2 protein and a fusion protein comprising a long alpha-helix (LAH) domain linked with four tandem M2e repeats have been shown to elicit corresponding HA- or M2e-specific humoral responses, with the LAH and 4M2e combination achieving 100% protection in murine models [25].

In addition to their role as vaccine vectors, probiotics also serve as potent adjuvants, significantly enhancing immune responsiveness to influenza vaccines. A placebo-controlled, randomized, double-blind clinical trial indicated that administration of a defined probiotic combination to subjects receiving influenza vaccination enhances immune activation, improves vaccine responsiveness, and promotes beneficial gut microbial composition [146]. Multiple animal studies and clinical trials have demonstrated that strains such as *Lactobacillus rhamnosus GG* (LGG), plant-derived *Lactobacillus*, and *Bifidobacterium* spp. effectively promote seroconversion and immunogenicity against influenza viruses, indicating that incorporating probiotics into vaccine formulations can substantially augment immunoprotection [85]. Notably, P18 peptides isolated from *Bacillus subtilis* demonstrate in vitro anti-influenza activity and confer protection comparable to oseltamivir (Tamiflu) in animal models. Such bioactive peptides with immunomodulatory properties, when integrated into conventional influenza vaccine platforms, may represent a pivotal breakthrough toward broad-spectrum anti-influenza immunoprophylaxis [147].

**Figure 2:**
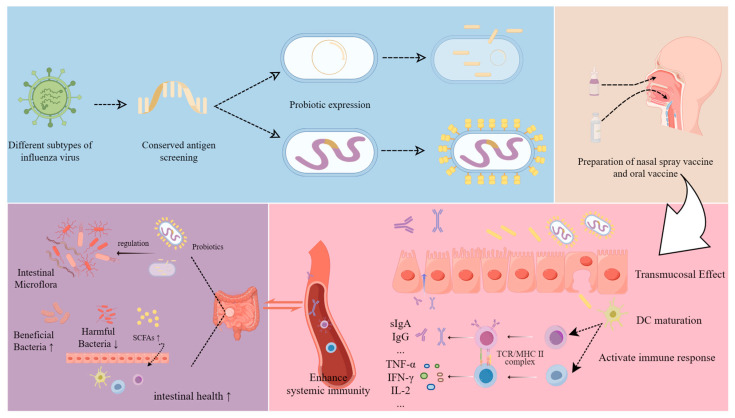
Construction and immune mechanism of a probiotic-based vaccine (created with FigDraw 2.0 (by figdraw.com)). Conserved antigens of influenza A virus (IAV) are selected and cloned into probiotic strains using genetic engineering techniques, enabling the probiotics to express these antigens either through secretion or on the bacterial surface. When administered intranasally or by inhalation, the vaccine activates mucosal and humoral immune responses against IAV and can also help regulate gut health.

A compelling observation from these studies is that probiotic-based influenza vaccines exhibit pronounced advantages in immunological durability, predominantly conferring protection against a limited number of specific viral strains (1–2), suggesting constrained antigenic breadth [148]. Although most published evidence is derived from mouse models or healthy human immunogenicity testing, no probiotic vaccine has demonstrated therapeutic benefit in influenza patients or reached approved clinical use. Nevertheless, probiotic vectors remain a promising adjunct to traditional vaccines due to their capability for intranasal or oral delivery, directly stimulating mucosal immunity in the respiratory and gastrointestinal tracts, with particular efficacy in inducing T lymphocyte-mediated responses and secretory IgA production (Figure 2). These attributes present innovative avenues for universal influenza vaccine development [122]. Moreover, probiotic-based vaccines offer benefits in manufacturing efficiency, cold chain requirements, and patient self-administration [149]. Despite encouraging preclinical findings, probiotic vector-based vaccine platforms remain at an early stage of development, prior to widespread clinical application. Further research is warranted to elucidate the effects of probiotic modification on host immune responses, the mechanisms underlying vector-induced immunogenicity, and strategies to enhance targeted delivery—such as surface molecule modifications, multivalent antigen presentation, and expansion of protective coverage—to optimize vaccine efficacy. And significant technical, safety, and regulatory challenges that must be addressed [150].

## 7. Discussion and Future Directions

The gut microbiota exerts extensive and multifaceted effects on host biological systems, particularly in immune regulation and metabolic homeostasis. Its ecological imbalance has been closely associated with the onset and progression of various infectious diseases, including influenza [151]. Evidence from animal models indicates that influenza infection can disrupt gut microbial composition, while alterations in the microbiota may, in turn, influence antiviral immune responses and disease severity. Notably, different influenza strains appear to induce distinct patterns of microbiota dysbiosis and host immune responses [152]. However, whether these effects are strain-specific or dependent on experimental models remains unclear.

Interventions targeting the gut microbiota—such as probiotics, fecal microbiota transplantation (FMT), and microbiota-derived metabolites—have demonstrated beneficial immunomodulatory and antiviral effects in preclinical studies. These approaches may help restore microbial homeostasis, reduce viral replication, lower the risk of secondary bacterial infections, and improve disease outcomes [153,154,155]. Both conventional probiotics (e.g., *Lactobacillus* and *Bifidobacterium*) and next-generation commensals (e.g., *Bacteroides*, *Prevotella*, and *Faecalibacterium*) have shown anti-influenza potential in preclinical settings, albeit through distinct mechanisms. In addition, recent studies have explored the potential of probiotics to enhance vaccine-induced immune responses, suggesting possible translational relevance in disease prevention and adjunctive therapy [24].

However, current mechanistic insights into the gut–lung axis—such as immune cell trafficking and short-chain fatty acid (SCFA)-mediated signaling—are predominantly derived from murine models. In contrast, human evidence remains limited and is largely observational. Only a small number of randomized controlled trials are currently available, and most focus on immunological or surrogate endpoints rather than clinically meaningful outcomes such as infection rates, symptom severity, or disease progression [39,100,145]. Furthermore, substantial heterogeneity in probiotic strains, study populations, and experimental designs limits comparability across studies and complicates the interpretation of their translational relevance. Overall, existing evidence provides a plausible mechanistic rationale for the role of gut microbiota and microbiota-targeted interventions in modulating host responses to influenza. Compared with conventional influenza prevention strategies, microbiota-based approaches may offer theoretical advantages, such as broad-spectrum immunomodulation and the potential to induce both mucosal and systemic immune responses. However, these proposed benefits should be interpreted with caution, given the current lack of robust clinical evidence.

Despite growing interest in this field, several key challenges remain. First, the definition and quantitative characterization of a “healthy” gut microbiota are not yet standardized, and complex interactions within the microbial ecosystem are not fully understood. Second, the immunomodulatory effects of probiotics are highly strain-specific, necessitating careful selection and validation of candidate strains in both preclinical and clinical studies. Third, clinical responses to microbiota-targeted interventions exhibit considerable inter-individual variability, likely influenced by host genetics, baseline health status, and initial microbiome composition. Large-scale, well-controlled interventional studies are therefore required to establish causal relationships between specific microbiota profiles and influenza-related outcomes in humans [156,157].

Future research should prioritize rigorously designed, adequately powered clinical trials to evaluate the efficacy of probiotics and other microbiota-targeted strategies in influenza prevention and treatment. Standardization of probiotic strain selection, dosing regimens, timing of intervention, and outcome measures will be essential to improve reproducibility. In addition, further investigation into host–microbiota interactions, including the identification of microbiota-derived biomarkers and functional metabolites, may facilitate patient stratification and the development of targeted therapeutic approaches.

Emerging strategies, such as engineered probiotics and microbiota-based therapeutics, warrant cautious exploration, with careful consideration of safety, regulatory, and translational challenges. Combination approaches—for example, probiotics in conjunction with influenza vaccines or antiviral agents—represent a promising direction but require robust clinical validation. Mechanistically, approaches involving SCFA-mediated dendritic cell maturation, engineered commensal delivery systems, and microbiome-guided precision interventions are being investigated to enhance vaccine efficacy and durability, particularly in the context of viral diversity and vulnerable populations [117,158]. Finally, the development of predictive models based on microbial diversity indices, key taxa abundance, and metabolite profiles may enable early identification of high-risk populations and support targeted preventive strategies [159]. As understanding of the microbiota–gut–lung axis continues to advance and translational applications evolve, microbiota-targeted interventions may emerge as complementary components of influenza control strategies.

## 8. Conclusions

This narrative review summarizes current evidence on the interactions between influenza virus infection, gut microbiota, and microbiota-mediated immune responses, as well as the potential role of probiotics in modulating these processes. Accumulating preclinical studies provide important mechanistic insights into the gut–lung axis and its role in antiviral immunity. However, translation of these findings into clinical benefit remains limited. To date, only a small number of randomized controlled trials have evaluated the effects of probiotics in the context of influenza or influenza-related outcomes, and these studies are limited in size and scope. Consequently, current evidence is insufficient to support definitive conclusions regarding the therapeutic or prophylactic efficacy of microbiota-targeted interventions in humans. Overall, while the gut microbiota represents a promising area of research in influenza, further well-designed clinical studies are required to establish its clinical relevance.

## Figures and Tables

**Table 1:** Representative probiotic strains and next-generation beneficial commensal bacteria evaluated in preclinical mechanistic studies of influenza infection.

Strain	Model	Measured Outcomes	Key Findings	Ref.
*Escherichia coli* Nissle 1917 ^1^	Mouse	Innate immunity markers	Activation of innate respiratory tract immunity	[22]
*Lactiplantibacillus plantarum* 16*/Lacticaseibacillus rhamnosus* P118 ^1^	Mouse	Viral load	Reduces viral load in the respiratory tract	[73]
*Lactobacillus plantarum* ^1^	Mouse	Viral replication	Inhibited viral replication in the lungs	[74]
*Lactobacillus plantarum* CNRZ1997 ^1^	Mouse	Viral replication	Inhibition of viral proliferation in the lungs	[75]
*Lactobacillus rhamnosus* CRL1505 ^1^	Mouse	Cytokines; coagulation markers	Modulated inflammatory and coagulation-related pathways	[76]
*Leuconostoc mesenteroides* (DRC1506 and 218) ^2^	Mouse	Survival	Improved survival after infection	[77]
*Lactobacillus plantarum* (330, CK10, and 920) ^1^	Mouse	Observational/clinical	Reduced the onset and duration of fever, runny nose, and cough	[78]
*Lacticaseibacillus rhamnosus* CCFM1279*/Limosilactobacillus reuteri* CCFM1145*/Lacticaseibacillus casei* CCFM1127 ^1^	Mouse	Viral replication	Inhibits viral replication and mitigates influenza-induced lung inflammation	[79]
*Lactobacillus delbrueckii* ssp. *bulgaricus* OLL1073R-1 ^1^	Mouse	IgA levels; cytokines	Increases IgA production and modulates cytokine responses	[80]
*Lactobacillus rhamnosus GG* ^1^	Mouse	Cellular immune response	Enhance the respiratory cell-mediated immune response	[81,82,83]
*Lactobacillus rhamnosus* LC705 ^1^	Cell	Inflammatory cytokines	Activated macrophage innate immune response	[82]
*Bifidobacterium animalis* subsp. *lactis* ^1^	Mouse	CD8^+^ T cell response; mucosal immunity	Increased the CD8^+^ T cell-mediated antiviral and systemic/mucosal immune responses	[84]
*Lactobacillus plantarum* AYA ^1^	Mouse	IgA levels	Increased mucosal IgA production	[85]
*Lactiplantibacillus plantarum* GUANKE ^1^	Mouse	Cytokines	Reduced pro-inflammatory cytokines levels	[86,87]
*Lactobacillus brevis* KB290 ^1^	Mouse	IFN-α; IgA	Increased IFN-α production and influenza-specific IgA	[88]
*Lactobacillus paracasei* CNCM I-1518 ^1^	Mouse	Lung immunity	Modulates pulmonary immune responses	[89]
*Lactobacillus paracasei* MCC1849 ^1^	Mouse	IgA; Tfh cells	Increased antigen-specific IgA and follicular helper T cells	[90]
*Lactobacillus casei* DK128 ^1^	Mouse	Immune activation	Activation innate immunity and adaptive Immunity responses	[91]
*Bifidobacterium bifidum* 379, 1, and 791 ^1^	Cell	Antiviral activity	Pronounced antiviral activity	[92]
*Lactobacillus pentosus strain* b240 ^1^	Mouse	Pulmonary IgA	Increased pulmonary IgA secretion	[93]
*Lactiplantibacillus pentoses* CCFM1227 ^1^	Mouse	Viral load; lung pathology	Reduced viral replication and lung immunopathology	[94]
*Lactobacillus reuteri* EHA2 ^1^	Mouse	SCFAs; IL-17	Increased SCFAs and reduced IL-17-mediated inflammation	[95]
*Bacteroides dorei* RX2020 ^2^	Mouse	IFN-β	Increased dendritic cell IFN-β production	[96]
*Limosilactobacillus reuteri* KBL346 ^1^	Mouse	Viral load; cytokines	Reduces pulmonary viral load and inflammatory cytokine	[97]
*Bacillus subtilis*-597 ^1^	Swine	Lung pathology	Reduced severity of lung lesions	[98]
*Faecalibacterium duncaniae* (A2-165 and I-4574) ^2^	Mouse	Viral load; inflammation	Reduces lung viral load and inflammation	[99]
*Lactobacillus acidophilus* L-92 ^1^	Mouse	Neutrophils; NK activity	Reduces neutrophils and boosts NK activity	[100]
*Lactobacillus gasseri* TMC0356 ^1^	Mouse	Viral load; immune response	Reduces viral load and enhance mucosal immune	[101,102]
*Lactobacillus* spp. ^1^	Mouse	IL-12, IgA, TNF-α, IL-6	Increased IL-12 and IgA; decreased proinflammatory cytokines	[103]
*Lactococcus lactis* subsp. *lactis* JCM5805 ^1^	Cell	Cytokines	Upregulation IFN-α and interferon-stimulated genes	[104]
*Lactobacillus pentosus* b240 ^1^	Mouse	Interferon-stimulated genes	Regulated pulmonary antiviral genes expression	[105]
*Prevotella copri* ^2^	Mouse	Survival	Improved survival and clinical outcomes	[106]
*Lactobacillus paracasei* CNCM I-1518 ^1^	Mouse	Secondary infection	Modulates pulmonary immunity, decreasing lung inflammatory cell accumulation, and accelerating viral clearance	[107]

^1^ Probiotic strains; ^2^ Next-generation beneficial commensal bacteria.

**Table 2:** Probiotic interventions evaluated in human immunogenicity and influenza vaccine response studies.

Strain	Population	Measured Outcomes	Key Findings	Ref.
*Bifidobacteria*	Human(vaccine)	Vaccine-specific antibody titers	Mitigated the detrimental effects of early-life antibiotics on vaccine immunogenicity	[108]
*Bifidobacterium longum* bv. *infantis* CCUG 52,486	Human(vaccine)	Vaccine-specific antibody titers	Increased vaccine-specific antibody responses	[109]
*Lactobacillus rhamnosus* GG	Human(vaccine)	rates of seroconversion after administration of LAIV	Improved influenza vaccine immunogenicity	[110]
*Bifidobacterium animalis* subsp. *lactis*	Human(vaccine)	IgG; IgA	Increased systemic/mucosal immune responses	[111]
*Lactobacillus paracasei* ssp. *paracasei*	Human(vaccine)	IgG; IgA	Increased systemic/mucosal immune responses	[111]
*Lactobacillus fermentum* CECT5716	Human(vaccine)	Th1 response; neutralizing antibodies	Increased Th1 response and virus-neutralizing antibodies	[112]
*Lactobacillus acidophilus* NCFM*/Bifidobacterium animalis subsplactis* Bi-07	Human(RTIs)	Observational/clinical	Reduces the incidence and duration of fever, cough, and runny nose.	[113]
*Bacillus coagulans* GBI-30, 6086	Human(clinical)	T cell response	Enhanced T cell-mediated immune response	[114]
*Bifidobacterium longum* BB536	Human(vaccine)	Serum IgA	Increased serum IgA levels	[115]
*Lactobacillus coryniformis* K8 CECT5711	Human(vaccine)	Vaccine response; RTI symptoms	Improved vaccine immunogenicity and reduced RTI symptoms	[116]
*Lactic acid bacterium* SANK70258	Human(Observational/clinical)	NK cell activity; sIgA	Increased NK cell activity and mucosal IgA levels	[117]

## Data Availability

All required data are available in the manuscript. Any additional data can be provided upon request.

## Abbreviations

The following abbreviations are used in this manuscript:

GALT	Gut-associated lymphoid tissue
SCFAs	Short-chain fatty acids
IAV	Influenza A virus
EcN	*Escherichia coli* Nissle 1917
FMT	Fecal microbiota transplantation
LPS	Lipopolysaccharide
